# Supplementary material for: Openspritzer: an open hardware pressure ejection system for reliably delivering picolitre volumes
Source: Sci Rep. 2017 May 19;7:2188. doi: 10.1038/s41598-017-02301-2 (PMC5438373; doi:10.1038/s41598-017-02301-2)
Supplement: Supplementary file 1 — Supplementary Information [file 41598_2017_2301_MOESM1_ESM.pdf]

Supplementary Material

Openspritzer: an open hardware pressure ejection system for reliably delivering picolitre volumes.

Forman, C.J.[1,5§], Tomes, H.[2], Mbobo, B.[2,3], Burman, R.J. [2], Jacobs, M.[3], Baden, T[1,4,5§], Raimondo, J.V.[2,5§]

Affiliations:
1) School of Life Sciences, University of Sussex, United Kingdom
2) Division of Physiology, Department of Human Biology, Neuroscience Institute and Institute of Infectious Disease and Molecular Medicine, Faculty of Health Sciences, University of Cape Town, Cape Town, South Africa
3) Division of Immunology, Department of Clinical Laboratory Science, Institute of Infectious Disease and Molecular Medicine, Faculty of Health Sciences, University of Cape Town, Cape Town, South Africa
4) Institute for Ophthalmic Research, University of Tuebingen, Germany
5) TReND in Africa gUG (www.TReNDinAfrica.org)

[§]Correspondence: C.J.F.: chrisforman@cantab.net, T.B.: t.baden@sussex.ac.uk, J.V.R.: joseph.raimondo@uct.ac.za

Command duration is correlated to dosage

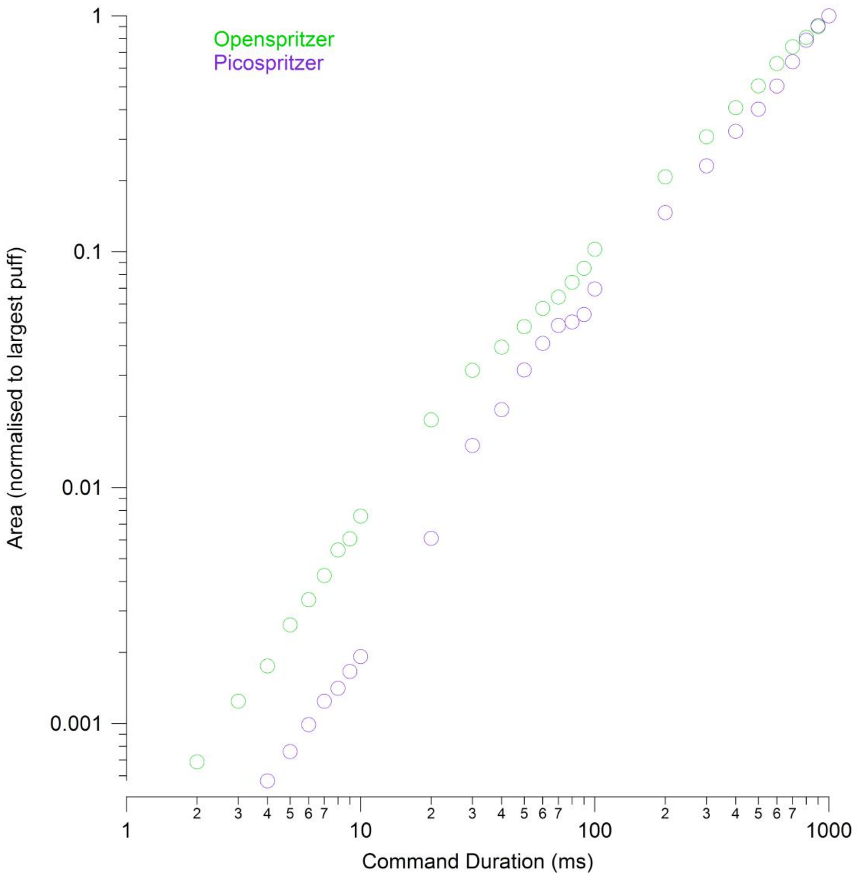

Supplementary Figure S1: The integrated area for each puff plotted against the command duration. The area under the puff profile is proportional to the dosage. Command duration and dosage are strongly correlated, regardless of the details of the puff profile over time.

Supporting Table S1: Comparison of commercial specifications for micro-injector systems

Name	Manufacturer	Method	Output Pressure	Volume/ Time range	TTL	Remote	Vacuum Holding	Dual	Program	Comments	Cost
Picospritzer III	Parker	Pressure	0 to 7 bar	2 ms to 999 m	✓	✓	✓	✓			£3400
IM-300	Narishige	Pressure	0 to 4 bar	10 ms to 327.66 s	✓	✓	✓	✓	✓		£4650
MINJ-D	Tritech	Pressure	0 to 7 bar	100 ms to 999 s	✓	✓	✓	✓	✓	Computer interface (+£320)	£3240
Picopump PV820	World Precision	Pressure	0 to 6 bar	10 ms to 10 s	✓	✓	✓	✓			£2340
Drummond	Nanoject III	Syringe		0.6 nl to 999.9 nl	✓	✓			✓	Datalogging,<br>Touch screen	£1900
MinJ-1	Tritech	Pressure	0 to 7 bar	100 ms to 15 s		✓	✓	✓		Time control (+£80)<br>Dual Pressure (+£240)<br>Vacuum Holding (+350)	£520
Femtojet 4i	Eppendorf	Pressure	0 to 6 bar	10 ms to 99.9<br>(10 ms steps)		✓		✓	✓	Integrated pressure<br>Semi-Automatic is Extra	£8100
Drummond	Nanoject II	Syringe		2.3 nl to 69 nl		✓					£1560
Openspritzer		Pressure	0 to 4 bar	2 ms to indefinite	✓	✓			✓		£360

The costs are indicative only and were acquired from 3rd party suppliers on 1st March 2017.

Openspritzer Construction

Overview

This document contains detailed assembly instructions and an operation manual for Openspritzer, and includes a parts list. The Arduino code and SCAD files for the exterior casing are provided and may be downloaded at http://raimondolab.com/Openspritzer/. The purpose of the device is to regulate the pressure and duration of a puff of compressed air. Typically the output port is connected to a glass puffer pipette which has been drawn into a sharp point with a narrow (<2-3 μm) diameter pore.

The device consists of a circuit board, a solenoid valve, a pressure regulator with a gauge and various interface components. Supplementary Fig. S2 shows the device without the covering lid and the solenoid displaced to expose the circuit board. The pressure regulator is mounted on the left and attached to the front panel *via* a retaining threaded ring that comes with the regulator. The circuit board is mounted onto attachment points on the floor of the box. It is situated next to the side wall containing the electronic interface components. There are two supports on which the solenoid can rest above the circuit board and an LED can be attached to a mount-point on the lid. There are also several retaining pillars which keep the solenoid in position. The lid has a block attached to it which acts to clamp the solenoid down when the box is closed.

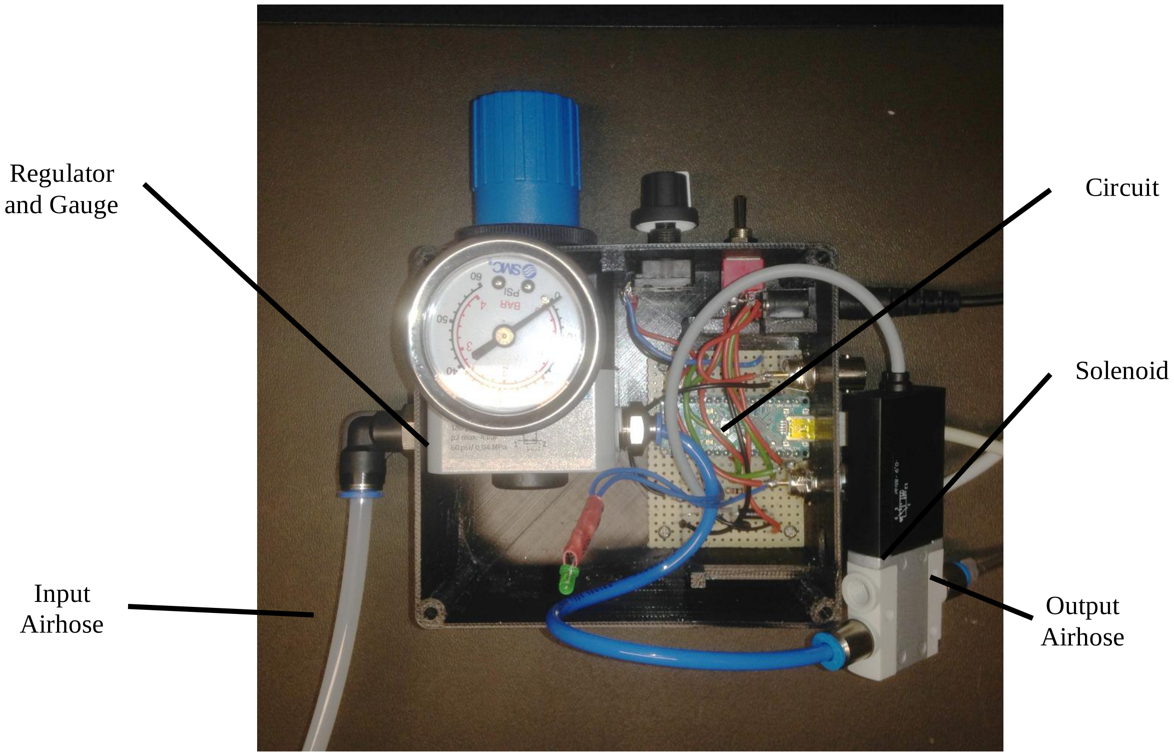

Supplementary Figure S2: Openspritzer consists of a pressure regulator, a solenoid and a control circuit. Its purpose is to control the strength and duration of a puff of compressed air, which is connected to a glass pipette that typically contains reagents for microinjection into biological samples.

Parts List

Supplementary Table 1 has an exhaustive list of parts, suppliers and prices, allowing calculation of the total cost of parts, which is between £320 and £360 at submission, depending on how much of the full system one chooses to include. Much of the circuitry e.g. the Arduino, footswitch and toggle switch is purely for convenience. A core functional system consists of the regulator, solenoid and a much simpler control circuit under TTL pulse control. The simpler minimal circuit is indicated in the construction instructions below, and consists of the branch of the circuit containing the transistor.

In addition, prices are highly variable depending on local availability of components. For example the single most expensive component when we assembled ours was the regulator which cost £180 at the time. However, we subsequently found the same regulator for $43 in a different country. If one is prepared to shop around, we estimate that a functional Openspritzer without add-ons could be constructed for as little as £175. If one has all the components it takes approx 3 hours to construct a full Openspritzer depending on experience, and excluding the print time for the case and lid.

Frequently components only come in bags of 10 or 50 so if one only intends to fabricate a single device there may be an additional overhead of extra parts, depending on the flexibility of your supplier. For example ours cost £360 for the parts actually included in the device, but we paid £495 in total and now have some spare components for additional units.

Supporting Table S2 Parts schedule for Openspritzer

Part	Manufacturer	Manufacturers Number	Remarks	Quantity	Essential	Component Price	System Price	Pack price	Unit
**Pneumatics**									
Solenoid (either 2 ms or 7 ms)	FESTO	MHE2-M1SH-3/2G-M7-K-RR	2 ms response + cable	1	Yes	£80.00	£80.00	£80.00	each
alternative		MHE2-M1H-3/2G-M7-K-RR	7 ms response + cable						
Precision pressure regulator	FESTO	LRP-1/4-4	0.05 to 4.0 bar	1	Yes	£180.00	£180.00	£180.00	each
Gauge	FESTO	MAP-40-4-1/8-EN	0.0 to 4.0 bar, 1/8 inch thread						
alternative	SMC	4K84P	0.0 to 4.0 bar, 1/8 inch thread	1	Yes	£30.00	£30.00	£30.00	each
Push-in (6 mm) to thread (1/4")	FESTO	QSL-1/4-6	6 mm to 1/4 inch	1	Yes	£2.83	£2.83	£28.30	Pack of ten
Push-in (4 mm) to thread (1/4")	FESTO	QS-1/4-4	4 mm to 1/4 inch	1	Yes	£1.76	£1.76	£17.60	Pack of ten
Push-in (4 mm) to thread (M7)	FESTO	QSM-M7-4-I	4 mm to M7 thread	2	Yes	£1.81	£3.62	£18.10	Pack of ten
4mm OD plastic tubing	FESTO	PUN-4x0.75	4 mm OD and 10 bar	12 cm	Yes	£0.09	£0.09	£35.50	50 m
**Electronics**									
Arduino Nano	Arduino		Arduino Nano	1	No	£4.00	£4.00	£4.00	each
Darlington pair NPN transistor	Fairchild	KSP 13BU	Ic = 500 mA, Vces = 30 V	1	Yes	£0.12	£0.12	£1.20	pack of ten
1 kΩ variable resistor	Vishay	249BBHS0XB25102KA	panel mount	1	No	£7.53	£7.53	£7.53	each
Knob for variable resistor	RS Online	299-4957	6mm shaft radius	1	No	£0.17	£0.17	£0.85	pack of five
DPDT switch	RS Online	734-7016	on-on panel mount	1	No	£1.13	£1.13	£5.65	pack of five
SPST footswitch	RS Online	335-319	push-to-contact	1	No	£13.03	£13.03	£13.03	each
24V dc desktop PSU 2.1 mm jack	RS Online	436-058	24 V dc 1 A	1	Yes	£17.91	£17.91	£17.91	each
2.1 mm right angle dc socket	RS Online	884-0957	2.1 mm right angle	1	Yes	£0.93	£0.93	£4.65	pack of five
Rect pin socket (through hole fem)	Digikey	SAM1222-01-ND	1x1	2	No	£0.81	£1.62	£1.62	each
Rect pin socket (through hole fem)	Digikey	S7038-ND	5x1	1	No	£0.65	£0.65	£0.65	each
Rect pin socket (through hole fem)	Digikey	3M9514-ND	3x1	1	No	£0.64	£0.64	£0.64	each
24 V Zener Diode	ON Semiconductor	1N5359BG	24 V	1	Yes	£2.30	£2.30	£2.30	each
1N4148 signal diode	Fairchild	1n4148		1	No	£0.01	£0.01	£0.01	each
3 mm LED	Digikey	350-1557-ND	3mm round, throughhole, grn	1	No	£0.95	£0.95	£0.95	each

Mount for 3 mm LED	Digikey	67-1330-ND	3mm panel mount		1	No	£0.26	£0.26	£0.26	each
220 Ω resistor	Digikey	CF14JT220RCT-ND	220 Ω through hole resistor		3	No	£0.10	£0.30	£0.30	each
1 kΩ resistor	Digikey	CF14JT1K00CT-ND	1 kΩ through hole resistor		3	Yes	£0.10	£0.30	£0.30	each
BNC connector male	RS Online	423-1927	50 Ω Cable mount solder		1	No	£3.26	£3.26	£3.26	each
BNC connectors female	Digikey	A97548-ND	Panel mount female		2	No	£1.60	£3.20	£3.20	each
Matrix board	RS Online	463-2819	2.5 mm pitch matrix board		1	Yes	£3.41	£3.41	£3.41	each
Red, black, blue and green wire	RS Online	331-862	20 AWG	50cm		No	£0.19	£0.19	£11.29	30 m roll
Cable shrink	RS Online	170-6377	Shrunk diameter 1.2 mm.	5cm		No	£0.04	£0.04	£1.02	1.2m

Mechanical

M3 x 24 screw	RS Online	293-325	M3 x 24 Hex socket cap		4	No	£0.27	£1.06	£13.29	box/50
M3 nut	RS Online	122-4400	M3 nuts		4	No	£0.02	£0.08	£1.89	bag/100
M2 x 5 machine screw	RS Online	489-9264	M2 x 5mm screw		4	No	£0.03	£0.12	£3.06	bag/100
Super glue	RS Online	330-4002	Super glue bottle			No			£4.05	bottle

**Totals**	**Cost**
Only essential parts	£321.26
Full system	£361.50
Full System + Spares	£495.82

Construction

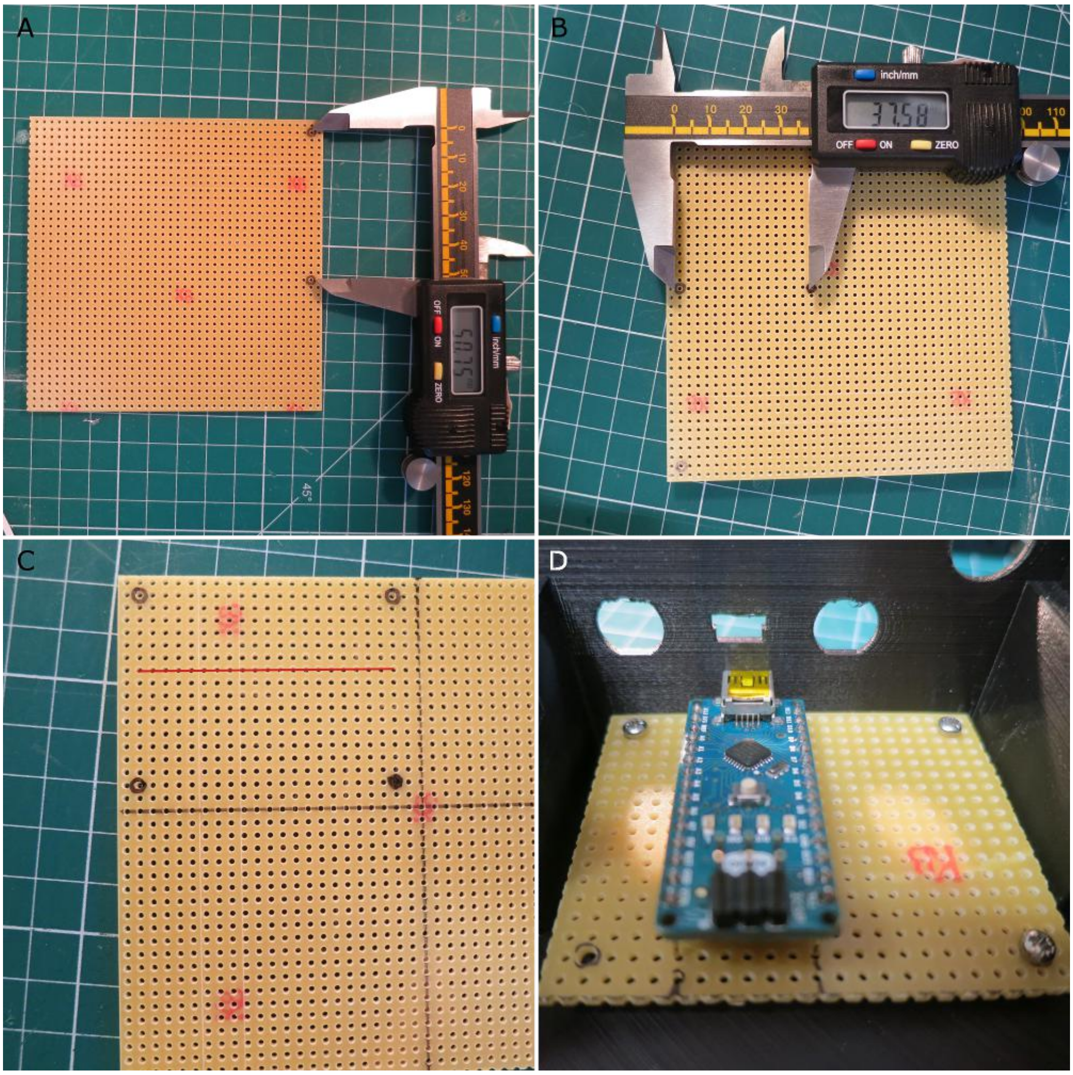

Supplementary Figure S3: A: Long edge of the matrix board measured between the mount point holes is 50.75 mm. B: The short edge is 37.6 mm. C: Ensure that the copper strips are parallel with the long edge and drill out the four mount point holes so they can comfortably contain an M2 screw without stressing the board. D: Test mount the board to correctly identify where to place the Arduino.

Matching the Circuit Board to the Box (Read BEFORE 3D printing the box!)

1. We used OpenSCAD (freely available at www.OpenSCAD.com) to design the box and lid respectively. The OpenSCAD script is provided. You may have to modify the measurements in the script to comply with the components that you have purchased.
2. In the SCAD file the box is aligned with the x-axis running parallel with the front and back panels of the box, and the y-axis parallel with the sides. The z-axis runs from bottom to top.
3. Prior to printing, ensure the mount points for the circuit board are spaced according to the holes on your matrix board (Fig. S3). In the OpenSCAD file you can adjust parameters *MountPointWidth* and *MountPointLength* to be the distance between the mounting holes in the board.
4. A pilot hole is incorporated down the centre of each mount point during printing and they are sufficiently tall to give clearance for any solder joints beneath the board. The mount points

are thick enough to accommodate an M2 screw, although excessive torque can shear them off.

5. The overall dimension of the circuit board should be approx 60 by 40 mm, which includes the overhang beyond the mount points.
6. The copper strips of the circuit board should be parallel with the long axis of the board (y-axis of the device).
7. The overhang beyond the mount points is specified with the *BoardEdgeX* and *BoardEdgeY* parameters, which are used to control how far the mount points are from the nearest walls.
8. Once the mount points are the right distance apart, their overall group position along the Y-axis can be adjusted by changing the parameter *BoardYPosition,* which moves all four mount points together.
9. The positions of the two BNC holes on the side panel are defined relative to the opening for the USB port.
10. The position of the USB port can be adjusted using *USBYOffset* and *USBZOffset*. Changing these values will move the entire group of holes associated with the circuit board – except the power supply hole.
11. *USBYOffset* is the distance in mm along the y-axis from the center of the opening for the USB port to the center of the mountpoint nearest to the side wall and the front wall.
12. The position of the solenoid retainer pillar may also need adjustment prior to printing depending on which version of the solenoid was purchased, and whether it has the integrated or detachable cable.
13. The OpenSCAD file referred to here employs the solenoid variant which has the cable permanently attached rather than the variant that employs a plug.
14. If you have the variant with the plug then you will have to adjust the locations of the front retainer pillars to fit the solenoid in firmly. The x position of the retainer can be adjusted using *SolRetOffsetX*.
15. Widen the circuit board holes corresponding to the mount points with a 2 mm drill bit.
16. Test mount the circuit board to identify which holes in the matrix board the arduino must be soldered to.

Constructing the circuit board.

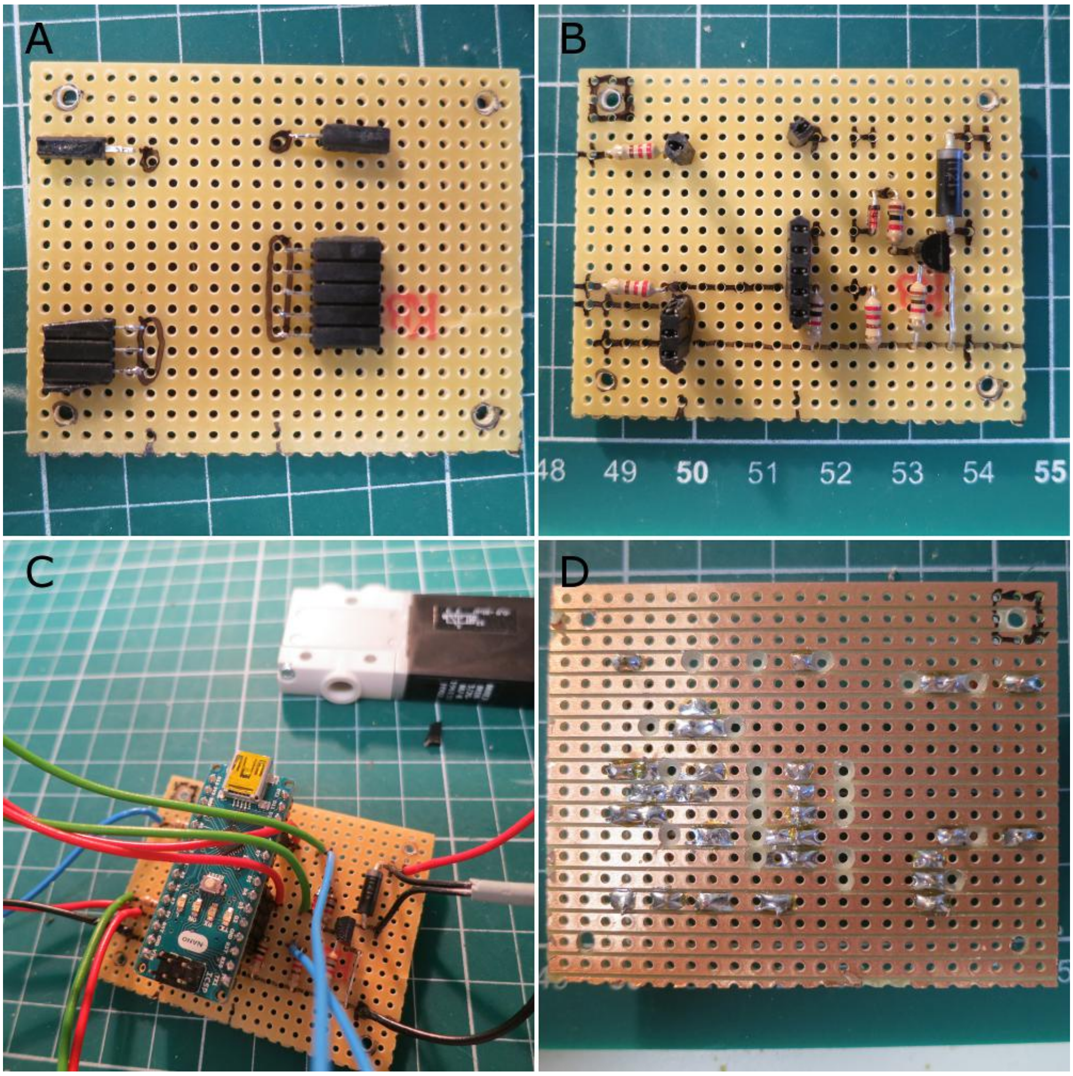

Supplementary Figure S4: A. Ensure the 5, 3, 1 and 1 terminal pin groups are in the correct place on the board. B. In the full circuit there are six resistors, two diodes and and a Darlington pair to solder into the correct positions. The marker pen indicates where connections exist under the board. C. Fly leads are attached wherever a short cross line is drawn on the circuit board. Ensure the solenoid cable is cut to an appropriate length before soldering (approx. 10 cm - see Fig. S2). D. Breaks in the copper strips beneath the board can be made using 2 mm drill bit rotated between finger and thumb.

1. Create four blocks of terminal strips containing 5, 3, 1 and 1 terminal pins, respectively.
2. Solder them in the positions indicated in Fig. S4A. It is important to get these in the right place so that Arduino matches up with the hole for the USB connector in the side panel.
3. The block of five sockets connects pins D5, D4, D3, D2 and GND to the board. The block of 3 connects 5V, BST and GND. The two single blocks connect D10 and A0. These are the only connections to the arduino that are needed.
4. Once the terminal blocks are in position all the components can be soldered to the board in the positions indicated in Fig. S4B. Note that if a stripped down circuit is being constructed (without the Arduino, LED or footswitch) then only the circuit downstream from the small

signal diode needs to be constructed – the branch containing the Darlington pair. In this case the 5V signal control signal must be connected directly to the signal diode.

5. The connections that are to be maintained by the copper strips under the board are indicated with the marker pen above the board. Wherever a small vertical crossline is included there will be a fly lead attached to the board for connection to the panel mounted or external components.
6. Fig. S4D shows where the copper strips need to be removed beneath the board. This can be achieved easily by rolling a 2 mm drill bit between finger and thumb and manually drilling part of the way into the board through the copper strip. There is a square drawn around the same attachment hole in Figs 4B and 4D to aid interpretation. Not all these holes need to be drilled out but isolated connection points help to avoid stray capacitances between long unused copper strips, which may affect circuit behaviour.
7. Fig. 4C shows where wires need to be attached to the board. Here we used 18 AWG which only just fit through the holes. In practice 20 AWG will suffice. In general red wires are attached to 5V or 24V supplies, black wires to the GND, and the blue and green wires to various intermediate potentials.
8. When connecting the solenoid to the circuit it is worth coarse positioning the circuit board and the solenoid in position within the box to ensure that the cable is cut to an appropriate length.
9. Once the basic circuit has been laid out and soldered, it is useful to ensure that each isolated connection point is indeed isolated using a multimeter to verify the connections, and isolate any short circuits from stray solder etc, before installation.

Assembling the Box.

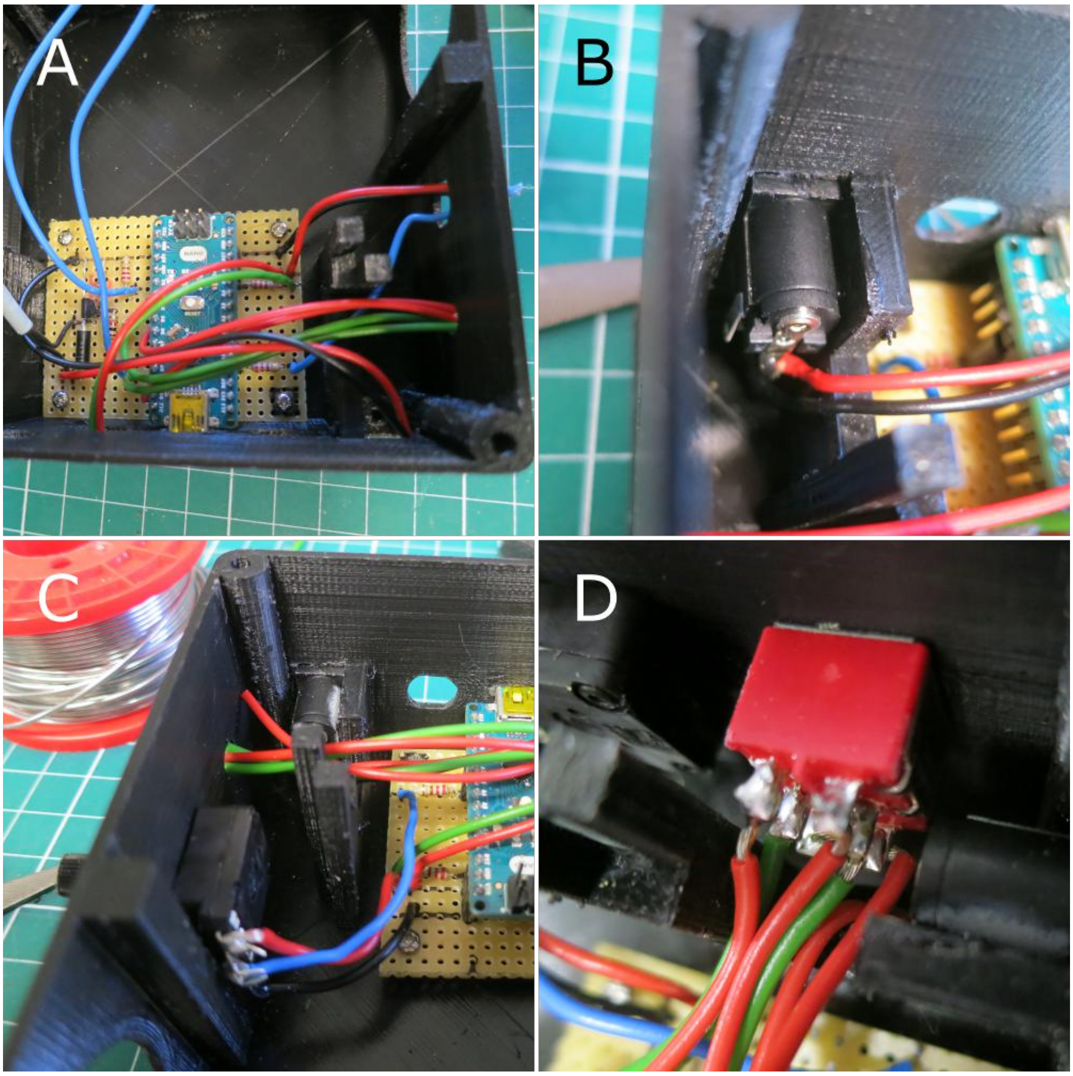

Supplementary Figure S5: A: The cable routes to their holes in the panel are shown, as well as a convenient method for determining a good length of cable. B: The power socket is superglued to the solenoid front support. C: Potentiometer wiring. D: DPDT wiring. The two lugs corresponding to the second ON state of the DPDT are wired together and the cable is routed to the BNC for the TTL pulse. Thus there are only five cables attached to the six pin DPDT. The sixth cable visible in image D is actually going to the 24 V powerline.

1. Screw the circuit board into position.
2. Cut each cable to an appropriate length. This can be achieved by routing the cables to the panel holes for which they are intended (as shown in Fig. S5A, and then cutting them on the other side of the panels. Leave the LED leads intact for the time being.
3. The cables for the 24 V power jack and the DPDT are routed through the gap in the front shelf underneath the solenoid.
4. The six upward facing pins on the Arduino should be cut off, desoldered or not installed in the first place as they interfere later with the pressure hoses and they are not used in this application. In the construction process during which the images were taken they were not

removed until the point at which this mechanical problem was first encountered. However, it would be easier to remove them at this point.

5. **Ensure that the Arduino is placed in position before soldering the panel components and mounting them on the panels.**
6. Mount the 24 V power socket before any of the other panel components (see Fig. S5B). Ensure that the socket can fit squarely into the opening before soldering it. You may need to adjust the size of the hole with a craft knife or a rasp. If you acquired the power socket in the parts list then there is a small plastic pin underneath the power socket which fits into the hole in the small panel beneath the power socket opening. Once you can install and remove the power socket easily then solder the cables into place ensuring the correct polarity. The central core of the socket should be positive. Then apply a small blob of superglue to the side of the power socket to hold it in place against the solenoid front shelf which is positioned perfectly square with the opening.
7. Next mount the potentiometer, by soldering the leads as shown in Fig. S5C and inserting the spindle through the panel hole, keeping the solder tags to the left hand side, by the regulator pillar. The potentiometer is held in place with a locking nut on the outside of the device. Cut the spindle to length with a hack saw and then attach a knob, as desired.
8. Fig. S5D shows the DPDT in place. To correctly connect this switch strip a single red cable to reveal about 1 cm of bare wire. The wire can be inserted simultaneously through the two lugs that belong to different sides of the DPDT but relate to the same switch state, thus shorting the two sides of the DPDT for that input. Solder the wire in place to both lugs. The other end of this wire will go to the BNC connector for an external TTL pulse, and will drive both the LED and transistor circuit simultaneously.
9. Next solder the green wires from the LED and transistor branches of the circuit to the pole of the DPDT on the two central lugs. The top two red wires for the other DPDT switch state are routed from the D10 and D5 Pins respectively. To match the code in the repository as it was at publication then make sure to connect these to their correct respective green wires so that pin 10 controls the LED and pin 5 controls the solenoid.
10. Once all six wires are in place the DPDT can be mounted on the panel, which requires use of the gap in the front solenoid shelf to install the switch. The orientation of the DPDT is set by a locking washer which has a small lug on it. Drill a small hole part way into the front wall panel at the appropriate distance directly below the hole for the switch. The lug on the locking washer sits into the hole and locks the orientation of the switch into a vertical plane. The locking nuts can then be screwed into place to hold it tight. The locking washer could be placed on the inside or the outside of the panel. Take care not to over-tighten the locking nuts as this risks shearing the collar on the switch.

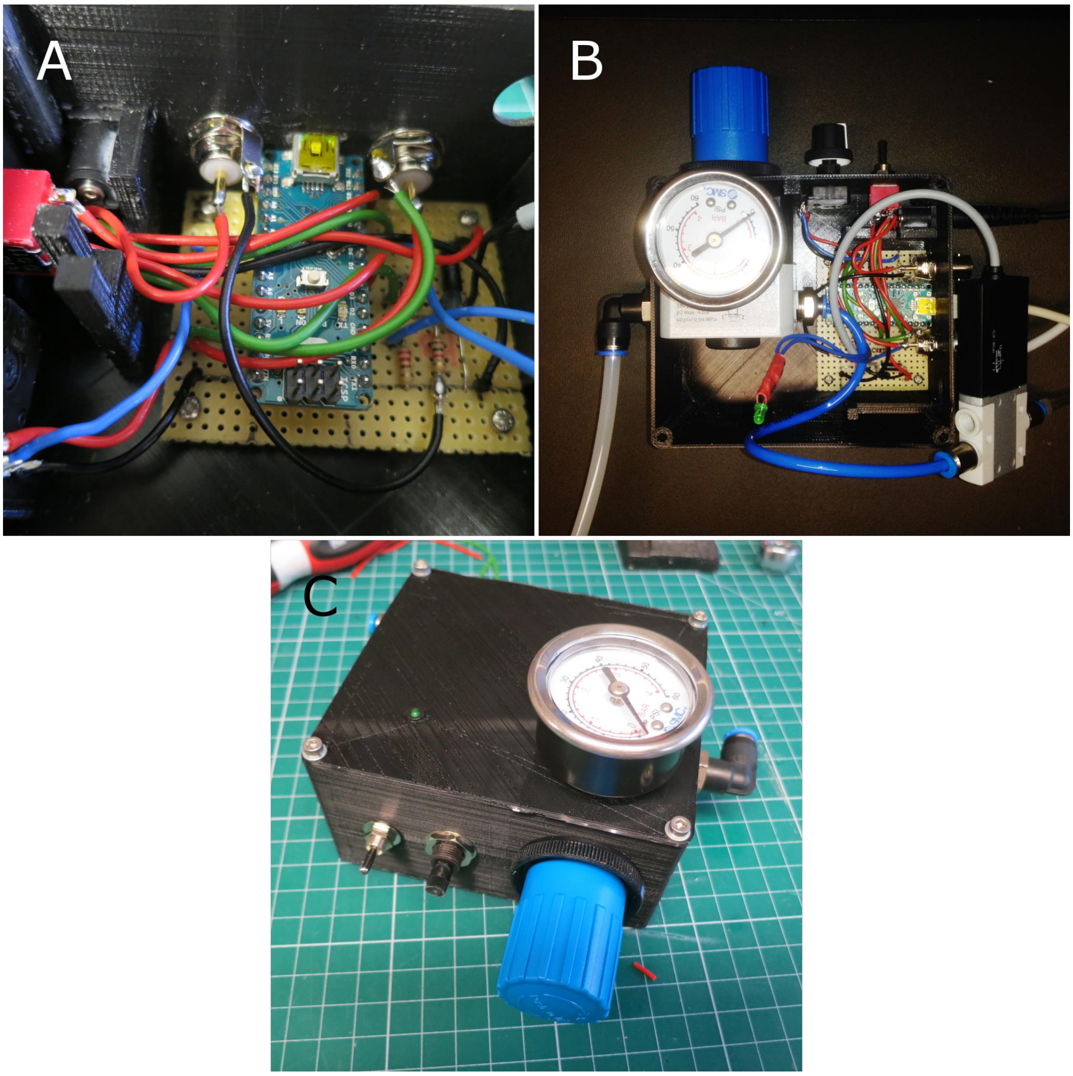

Supplementary Figure S6: A: The core pin of the TTL BNC is connected to the second ON position of the DPDT and the ground tag to the ground of the circuit. The core pin of the footswitch BNC is connected to 5 V and the ground tag is connected via a resistor to Pin D2 for monitoring. There is also a pull down resistor in the circuit to ensure an off reading when the switch is not closed. B: A workable arrangement of hoses, solenoid cable and LED wires. The hose connects port 2 of the regulator to port 1 on the solenoid, and the LED is the final component to be soldered into place. C: The final addition is the gauge which screws directly into the regulator through the lid.

11. The next step is to install the BNC connector for the external TTL pulse. The connectors are easily placed into the hole and, locked in place with a locking nut. Care must be taken to include the solder tag for grounding the BNC. The shorted lugs on the DPDT should be connected to the central pin of the BNC and the grounding tag should be connected to the ground on the circuit board. This can be achieved by soldering it directly to the end of the resistor as shown in Fig. S6A. Or you could install this wire earlier, along with all the others, from any spare hole on the ground line.
12. The second BNC is for the foot switch. The 5 V line should be connected to the central pin and the grounding tag should be connected to the green wire shown, which connects via a resistor to pin D2 which monitors the foot switch.
13. Fig. S6B indicates how to install the regulator and solenoid. First the locking ring is removed from the regulator and the knob is fed through the large circular aperture ensuring that port 1 is

facing to the left panel and the gauge hole is exposed on the top surface. Although it is shown in the image, do not attach the gauge at this time.

14. A 1/4 inch thread to 4 mm push fit adaptor is placed in port 2 of the regulator to which a 10 cm blue hose is then attached.
15. The hose is routed above the circuit board and back out to the rest of the box, ensuring that it passes around the back of the solenoid cable. The solenoid can then be placed into position ensuring that port two is next to the output hole in the panel.
16. The LED wires should come up inside the solenoid cable. Place heat shrink tubing on to the LED leads before soldering the LED, ensuring the correct polarity of the LED.
17. After the LED is soldered in place, cover the contacts with the heat shrinking tubing and use a heat gun or soldering iron to shrink the tubing.
18. The blue hose can then be inserted into port 1 of the solenoid.
19. Remove the blocking screw from the top of the regulator unblocking the aperture for the gauge.
20. An L-shaped adaptor is inserted into port 1 of the regulator through the left hand side panel.
21. An M7-threaded to 4mm push fit adaptor is insert into port 2 of the solenoid through the right hand side panel.
22. The lid is then placed coarsely in position, and the LED mount is placed in the hole. The LED is placed in turn into the mount, which should lock it in place.
23. If you purchased the SMC gauge then use a hack saw to remove the two *side* screws from the gauge. Don’t remove the central brass screw that is the pressure input to the gauge.
24. You can then place the gauge though the lid and screw it into the regulator.
25. To screw the lid into place you must first insert a hex-nut into the holes on the base of the box. You can do this by part screwing the nut onto a second screw and pushing it up inside, and then unscrew the screw.
26. Once the nut is in the hole (even partially) you can draw it up inside from above with the screws that hold the lid in place.

Programming the Arduino on board Openspritzer

1. Once Openspritzer is assembled the Arduino must be programmed.
2. Download and install Arduino programming environment on your computer (www.Arduino.org).
3. Open the .ino code for Openspritzer which is included in the Supplementary Materials.
4. Compile and upload code to the Openspritzer by connecting the USB port of Openspritzer to the USB port of your computer and clicking on the "Upload" icon in the top left corner of the screen.
5. Code can be modified and uploaded as many times as you like. The way the Openspritzer code has been constructed allows simple modification of key parameters such as the base unit of time. These are all included at the beginning of the code for ease of reference and are listed in supporting table 3.
6. **IMPORTANT**: The first time that the Arduino is used the POT_VALUE_LOW and POT_VALUE_HIGH parameters should be measured. A Serial.println statement is included in the code to allow monitoring of this value over the serial port.
7. To measure these values open the serial port monitoring window in the Arduino programming environment and then sweep the potentiometer from low to high.
8. The two parameters POT_VALUE_HIGH and POT_VALUE_LOW should be initialised to the highest values and lowest values observed in the monitoring window output.
9. This is used in the calculations of the pulse duration and will vary from Openspritzer to Openspritzer depending on the precise value of the resistances used in the construction of the device.

Operating Openspritzer

1. Connect an air compressor to the input port 1 of the regulator via the L-shaped adaptor.
2. The output airhose must be connected to the application.
3. Connect the 24 V power supply to operate the solenoid.
4. It is not necessary to use the arduino to operate the solenoid if you have a convenient method for producing 5 V TTL pulses, such as PulseQ electrophysiology package (Funetics) running on Igor Pro (Wavemetrics) in conjunction with the ITC 1600.
5. To use TTL pulses ensure the DPDT switch is in the TTL position. Whenever the 5 V line is high the solenoid will open and release compressed air from the output valve.
6. A good pressure for sharp crisp pulses, which helps the solenoid to close again, is 1.4 bar (20 psi). **Important**: When first connecting high-pressure air to Openspritzer, e.g. from a compressor or air-tank, make sure that the Openspritzer gauge is near zero. A too-high line pressure (>>1.4 bar) risks irreversibly damaging the solenoid.
7. For convenience in darkened circumstances the LED will also illuminate whenever the 5 V line is high.
8. Using the Arduino to drive the puffer allows operation with the external footswitch.
9. To operate with a footswitch the DPDT must be placed in Arduino mode and a 5 V power supply provided to the Arduino via the USB port.
10. The 5 V power supply can be any standard USB power supply and need not be a computer.
11. Attaching a computer at operation time allows for dynamic reprogramming of the Arduino during experimentation, which can be very useful. E.g. resetting the base time unit.
12. When the Arduino is first powered on the Openspritzer enters the potentiometer monitoring loop to intialise the pulse duration. During this process The LED lights up for half a second and then blinks to indicate the pulse duration. Such behaviour also indicates that the code is running properly.
13. To operate the unit press the foot-button and after 400 milliseconds a puff of a predefined duration will occur.
14. The LED will illuminate during the puff to give visual confirmation that the puff has occurred.
15. The duration of the puff can be changed by adjusting the potentiometer, which selects the integer number of times that the base time unit is repeated during the puff.
16. When the potentiometer is changed Openspritzer goes into a monitoring mode during which time the LED is continuously lit.
17. When the potentiometer stops changing Openspritzer blinks the LED to indicate the new time duration.
18. For example: if seven blinks occur after leaving the monitoring mode and the base time unit is set to 10 ms in the software then the duration of the pulse will be 7 x 10 ms = 70 ms.
19. If the button is clicked twice within the first four hundred milliseconds after the first click then a pre-programmed sequence of puffs will occur.
20. The default sequence is 28 pulses ranging from 2 ms up to 1000 ms and spaced two seconds apart. This is the method used to generate the puff sequences used in figure 2 of the main paper.

21. The pulse sequence can be interrupted by clicking the foot button during the sequence upon which occurrence the led will blink rapidly and then revert to stand-by.

Openspritzer Code Parameters

Keyword	Default Setting	Meaning
TIMEUNIT	100	Base time unit in milliseconds.
POT_VALUE_LOW	0	Analogread of lowest setting of potentiometer (measured).
POT_VALUE_HIGH	1023	Analogread of highest setting of potentiometer (measured).
SENSITIVITY	10	Number of settings of potentiometer.
POT_NOISE	5	Range of fluctuations in the pot_value when static.
STABILITY_TIME	500	Number of milliseconds stability required to accept a new pot_value.
MONITOR_DELAY	100	Delay between loops of the analog monitoring mode.
PAUSE_BEFORE_BLINK	500	Milliseconds delay before blinks occur after exiting analog monitoring mode.
BLINK_ON_DURATION	100	Duration for which the LED is on during the blinking phase.
BLINK_OFF_DURATION	250	Duration between blinks in the blink phase.
NUM_PROGRAMMED_PULSES	19	Number of puffs in hard coded sequence.
PROGRAMMED_SEQUENCE[19]	**Error!**	Puff duration sequence in ms.
PROGRAMMED_PULSE_DELAY	2000	Delay between puffs in programmed sequence.
MONITOR_FOOTSWITCH_PERIOD	400	Period to monitor for second button click.
FOOTBUTTONDELAY	1000	Main loop time delay allows button release.

Supplementary Table S3: Main user adjustable parameters of Openspritzer software

1. Supplementary Table S3 indicates the main parameters in the code which can be adjusted by the user and uploaded to Openspritzer to allow customised behaviour.

2. The user is free to write any code they wish to monitor the foot button, read the potentiometer, blink the LED or operate the solenoid. This code included alongside this paper was found to be convenient when used to record the data in the paper.
3. The monitoring algorithm relies on the POT_NOISE parameter to decide whether or not the potentiometer has settled.
4. To set POT_NOISE, monitor the serial port output during the analog monitoring procedure and find the POT_VALUE measured by the analogread command. POT_NOISE should be larger than any fluctuations in the pot_value when it is not being altered.
5. During testing, depending on which power supply was used, it was found that a reasonable value for POT_NOISE was 5.
6. The SENSITIVITY parameter increases the number of settings that are available by adjusting the potentiometer, which could be used to yield higher precision in the duration, or increase the overall range of durations.
7. If SENSITIVITY is set too high then the range of potentiometer values assigned to each setting will become smaller than POT_NOISE and the monitoring algorithm will never exit.
8. For example, typically there are 1024 values available for the pot_value, so a SENSIVITY of 10 will give 113 different values that correspond to each setting.
9. The lowest position of the potentiometer is defined to give yield a multiplier setting of 1 and the highest setting yields SENSITIVITY.
10. Depending on the linearity of the potentiometer the other values will be dispersed evenly.
11. A reasonable size range of pot_values should be assigned to each integer setting to give the user a chance to find that value.
12. In practice a SENSITIVITY of 10 settings was found to be adequate and easy to gauge the right rotation of the potentiometer.
13. The probability of mis-counting the confirmation pulses become much higher over ten pulses.
